# *Voices of conference attendees*: how should future hybrid conferences be designed?

**DOI:** 10.1186/s12909-024-05351-z

**Published:** 2024-04-09

**Authors:** Sai Sreenidhi Ram, Daniel Stricker, Carine Pannetier, Nathalie Tabin, Richard W Costello, Daiana Stolz, Kevin W Eva, Sören Huwendiek

**Affiliations:** 1 Institute for Medical Education, Department for Assessment and Evaluation, University of Bern, Mittelstrasse 43, 3012 Bern, Switzerland; 2 Graduate School for Health Sciences (GHS), University of Bern, Bern, Switzerland; 3 European Respiratory Society, Lausanne, Switzerland; 4 Department of Respiratory Medicine, Royal College of Surgeons, Dublin, Ireland; 5 The Clinics of Respiratory Medicine and Pulmonary Cell Research, University Hospital Basel, Basel, Switzerland; 6 Clinic of Respiratory Medicine, Faculty of Medicine, University of Freiburg, Freiburg, Germany; 7 Centre for Health Education Scholarship, University of British Columbia, Vancouver, Canada

**Keywords:** Virtual conference, Hybrid conference, In-person conference

## Abstract

**Background:**

With conference attendees having expressed preference for hybrid meeting formats (containing both in-person and virtual components), organisers are challenged to find the best combination of events for academic meetings. Better understanding what attendees prioritise in a hybrid conference should allow better planning and need fulfilment.

**Methods:**

An online survey with closed and open-ended questions was distributed to registrants of an international virtual conference. Responses were then submitted to descriptive statistical analysis and directed content analysis.

**Results:**

823 surveys (Response Rate = 4.9%) were received. Of the 813 who expressed a preference, 56.9% (*N* = 463) desired hybrid conference formats in the future, 32.0% (*N* = 260) preferred in-person conferences and 11.1% (*N* = 90) preferred virtual conferences. Presuming a hybrid meeting could be adopted, 67.4% (461/684) preferred that virtual sessions take place both during the in-person conference and be spread throughout the year. To optimise in-person components of hybrid conferences, recommendations received from 503 respondents included: prioritising clinical skills sessions (26.2%, *N* = 132), live international expert presentations and discussions (15.7%, *N* = 79) and interaction between delegates (13.5%, *N* = 68). To optimise virtual components, recommendations received from 486 respondents included: prioritising a live streaming platform with international experts’ presentations and discussions (24.3%, *N* = 118), clinical case discussions (19.8%, *N* = 96) and clinical update sessions (10.1%, *N* = 49).

**Conclusions:**

Attendees envision hybrid conferences in which organisers can enable the vital interaction between individuals during an in-person component (e.g., networking, viewing and improving clinical skills) while accessing virtual content at their convenience (e.g., online expert presentations with latest advancements, clinical case discussions and debates). Having accessible virtual sessions throughout the year, as well as live streaming during the in-person component of hybrid conferences, allows for opportunity to prolong learning beyond the conference days.

**Supplementary Information:**

The online version contains supplementary material available at 10.1186/s12909-024-05351-z.

## Background

In-person meetings have long offered important opportunities for professional development for clinicians and academics alike by promoting research, education, and career advancement. Among other things, such events have enabled networking and the introduction of new technologies and techniques into practice [1]. Research has suggested that in-person dialogue and debate through lectures, poster sessions and roundtable discussions are keys to conference success [2]. In addition, meeting other researchers, maintaining networks [3, 4], and discovering career opportunities [5, 6], are all important to attendees. Further, collaborative exchange between multidisciplinary members has demonstrated positive impacts on collaborative outcomes [7]. Such activity, however, is not without cost as meetings require effort (e.g., travel to conference venues, disruption to work and personal lives, and complex logistical planning for parents or caregivers) as well as money to cover registration, airfare, mileage, accommodation, and meals [8].

Those challenges amplified in response to the pandemic, as many conferences were forced online [9], requiring organisers and delegates alike to adapt to virtual environments. Research conducted on the transition has shown that motivations for conference attendance differ between in-person and virtual conferences, demanding a re-think about delegate priorities [10]. Virtual conference experiences have generally been reported as satisfactory [11] due to their being far more accessible, inclusive, and sustainable compared to in-person formats [12]. They appear to enable new learning [11] and allow populations with fewer resources to participate, thereby supporting equity, diversity and inclusion efforts [12]. Attending from the comfort of one’s own home or office [6] fosters a comfortable environment, but creating opportunities for interacting, networking and collaborating in a virtual format is challenging as chat boxes are a pale alternative to face-to-face discussion [13]. However, overwhelming digital-meeting fatigue, impersonal interactions and challenging time zones [14] also present challenges. Furthermore, online experiences have not been able to substitute for the hands-on learning via direct interaction with senior colleagues [15]. This is particularly notable within medical skills training (e.g., surgical procedures) [16]. That said, the benefits listed along with reduction in environmental impact [17] all suggest that virtual interactions are here to stay rather than reflecting a transitory adjustment.

In compromise, as the pandemic has subsided, hybrid conferences have become more prominent [18] and pressure on organisers to maintain hybrid formats is mounting as the majority of attendees now express preference for hybrid conferences [19]. That is, it has been shown that the majority of conference delegates prefer hybrid formats [15] because such meetings combine the advantages of in-person and virtual meetings [20]. For example, in-person conferences allow better interactivity with other delegates, better networking opportunities and better concentration whereas virtual conference formats were preferred for being time saving, cheaper and safer during the pandemic while also being more globally inclusive [10]. Hence, the combination of both allows for catering to diverse attendees’ needs.

As with anything, however, there are many ways in which a seemingly straightforward idea like “hybrid conferences” can be operationalised, requiring greater clarity regarding what organisers should prioritise as they continue to seek innovative ways to strengthen learning, global accessibility, and flexibility [10]. In other words, although there is a preference for hybrid formats, it is to date unclear how to optimise hybrid conferences to meet attendees’ needs. Suggestions from recent literature include that hybrid conferences may take the form of local in-person hubs, with a small number of participants meeting in parallel with online and virtual activities that include lectures to wider audiences [20]. While social interactions are more efficient during in-person gatherings, enabling virtual interaction with a wider array of individuals is more challenging. Ideas to address this include creating opportunities for social interactions through a virtual portal in which speakers can engage in discussions with delegates [20]. How to manage such innovations in the context of large-scale conferences, however, as well as what attendees would prioritise has not yet been published.

To address this gap, we surveyed conference delegates regarding how future hybrid conferences should be designed. Our main research question was “What are conference attendees’ preferences for in-person versus virtual components of hybrid conferences?” We triangulate on this question by asking meeting attendees about their preferences both in general terms and by inquiring about what could have been improved in the context of a large-scale virtual conference. By conducting this research, we aimed to provide insights into ways to increase the overall utility of academic conferences by providing guidance regarding what should be prioritised by meeting organisers.

## Methods

### Context

This study was conducted in conjunction with the second virtual European Respiratory Society (ERS) annual congress, which took place in September 2021. 16,888 international delegates registered for the meeting, which occurred face-to-face until 2019. The conference attracts individuals with an interest in respiratory medicine from a variety of disciplines and career stages, coming together to present and discuss the latest scientific and clinical advances in the field. Traditionally, the conference included expert presentations with structured sessions for knowledge, clinical skills and networking. When the COVID-19 pandemic emerged, the ERS congress moved to a virtual format for its September 2020 meeting. That virtual conference included a live online streaming platform that was structured similar to news channels (i.e., attendees could stream a variety of “programmes”) that included presentations delivered by the world’s respiratory experts to enable discussion of the latest scientific and clinical advances across the field of respiratory medicine. In addition to providing knowledge updates, clinical debates and case discussions were encouraged. In addition, attendees were given the opportunity to virtually present their own local, regional and international research with experts chairing each session.

### Study design

A survey was developed and distributed that was comprised of 2 parts: (1) overall motivations regarding why participants attend conferences; and (2) preferences for conference format and optimisation. The first part is largely a replication of previous work while the second is the primary focus of this study (*see Appendix 1*). Both sections were designed using AMEE Guide No. 87 [21] with full details on how the guidelines were followed outlined in the Appendix of Ram et al. [19].

In particular, six main steps were followed. Summarised with particular attention to their relevance for this study, they consisted of the following:

(1) Literature review and alignment with previous research: Using prior research and the study results reported by Ram et al. [19], we knew that the majority of respondents would like to see hybrid conferences in the future and we were able to make adjustments to prioritise focus on what particular components of virtual and in-person conferences would be considered optimal by attendees.

(2) Interviews to understand how others conceptualise the concept: SR had previously conducted semi-structured interviews with thirteen ERS stakeholders who had extensive conference attendance experience [19]. They were asked what they believed motivates conference attendance. A theme extracted from that work pertained to convenience, so we added questions focussed on barriers to in-person attendance.

(3) Findings synthesis and (4) Question development: Our previous success with the online survey format and inclusion of both closed questions and free text questions led us to adopt a similar structure for this work. Mandatory closed questions included aspects of virtual and in-person conferences that make them successful, delegate satisfaction with a virtual conference, and format preferences. Open free-text questions were used to gain a more descriptive account of respondents’ viewpoints regarding what should be prioritised during in-person and virtual components of hybrid conferences, improvements that could be made to virtual only conferences and barriers to in-person conference attendance. Demographic variables included age, gender, country, workplace and professional role.

(5) Expert validation: ERS educational chair members were invited to review the survey and refine any items they felt required clarification.

(6) Pilot testing: Three cognitive interviews were conducted with conference attendees from various disciplines and who were at different stages of their career. This was done to check whether all the items were understandable and to assess how long the survey would take to complete online.

### Data collection

SurveyMonkey (https://www.surveymonkey.com) was used to obtain informed consent from participants, and to execute the study. 16,888 attendees were invited to participate, via email, after the conference. Invitations included a brief description of the study, and a link to the survey with consent form included. Two reminder emails were sent over the course of a month with an incentive to win a free registration to the ERS Congress 2022. After gaining informed consent from participants, measures were taken to ensure confidentiality and anonymity of the data and by removing any identifying information from participant responses.

### Data analysis

Closed questions were summarised through descriptive statistics and open free-text questions were analysed using directed content analysis [22]. The latter involved extracting keywords from the literature review that informed stage 1 of survey development. They predominantly fell into two categories: in-person attendance challenges and virtual conference challenges. Namely, for in-person attendance challenges, *cost*, *conference registration*, *travel effort*, *language difficulties*, *time commitment*, and *accommodation* were all issues that were used to define the focus of any given comment; for virtual conference challenges, *internet connection*, *virtual networking*, and *time zones* were known to be key issues. These served as a starting point with additional codes being added as the analytic process continued whenever a substantive issue was raised that could not be coded using one or more of these key words. That is, any text that could not be categorised with the initial coding scheme was used to develop a new code that was then added to the code book.

Chi-squared analyses were conducted to compare the distribution of responses when participants were asked to comment on in-person versus virtual components of hybrid conferences.

## Results

823 attendees (Response Rate = 4.9%) completed the survey. 40.5% (*N* = 333) reported being male, 39.9% (*N* = 329) reported being female, 0.4% (*N* = 3) preferred not to say and 19.2% (*N* = 158) did not answer. Age was normally distributed with a peak in the 41–45-years-old range (*Appendix 2*). The modal workplace (39.3%, *N* = 261) was a university hospital (*Appendix 3*). 75.0% (*N* = 617) had attended the previous ERS virtual congress in 2020. 27.8% (*N* = 229) of participants had never attended an ERS congress (i.e., either a past in-person congress or the virtual ERS Congress in 2020).

From a total of 665 attendees who indicated their geographic location, 56.8% were from Europe (*N* = 378), 26.3% were from Asia (*N* = 175), 6.2% were from Africa (*N* = 41), 4.5% were from South America (*N* = 30), 4.2% were from North America (*N* = 28), and 2.0% were from Oceania (*N* = 13). While no demographics are available for all of the 2021 ERS congress attendees, these proportions compare well to those of a previous conference [19].

### Conference preferences

Consistent with our previous work, the majority − 56.9% (463/813) - of respondents claimed they would prefer conferences to use a hybrid format in the future. 32.0% (260/813) preferred in-person meetings and 11.1% (90/813) preferred virtual formats alone. Barriers to attendance at in-person conferences were primarily cost related (reflecting 74.0% (361/488) of the reasons given for difficulty attending in-person). 21.5% (105/488) of the barriers expressed related to travel challenges (including the time required) and a small minority mentioned other things such as difficulty getting out of clinical duties and language barriers.

### Optimising in-person components of hybrid conferences

503 free-text responses were received to the question: “We are thinking of moving to Hybrid conferences (combination of virtual and in-person components) for the future. What would you like to see in the in-person component?” In descending order of prevalence, 132 (26.2%) indicated a desire for clinical skills sessions, 79 (15.7%) wanted experts’ presentations and discussions, and 68 (13.5%) mentioned opportunities for interaction between all members (e.g., attendees, speakers, patients). Full details of the direct content analysis codes and their frequencies for in-person components of hybrid conferences are included in Table 1. 21.9% of participants (180/823) selected a preference for the in-person component to be held over *Friday-Saturday-Sunday*, closely followed by a preference for *Monday-Tuesday-Wednesday*, which was chosen by 20.1% (165/823) of participants, and *Thursday-Friday-Saturday*, which was chosen by 19.8% (163/823) of participants.

### Optimising virtual components of hybrid conferences

When respondents were asked to reflect on their preferences for the virtual components of hybrid conferences, 67.4% (461/684) indicated desiring virtual sessions both during the in-person congress and spread throughout the year. 18.0% (123/684) preferred virtual sessions only during the in-person event and 14.6% (100/684) preferred virtual sessions throughout the year rather than during the in-person event.

486 free-text responses were received to the question: “We are thinking of moving to Hybrid conferences (combination of virtual and in-person components) for the future. What would you like to see in the virtual component?” In descending order of preference, 118 (24.3%) indicated a desire for live streaming of experts’ presentations and discussions; 96 (19.8%) wanted virtual clinical case discussions; and 49 (10.1%) mentioned knowledge update sessions. Full details of the direct content analysis codes and their frequencies for virtual components of hybrid conferences are included in Table 1.

Chi-squared analyses conducted on codes that are applicable to both in-person and virtual conference components showed that the preference for “Clinical skills sessions” was mentioned a greater proportion of the time in the context of in-person components whereas “Clinical case discussions” and “Poster and oral presentation sessions” was mentioned a greater proportion of the time in the context of virtual components of hybrid conferences.

**Table 1:** Attendees’ preferences for both in-person and virtual components of hybrid conferences

	In-person Component (Total *N* = 503)	Virtual Component (Total *N* = 486)
Code from directed content analysis	% (N)*	% (N)*
Clinical skills sessions	26.2** (*N* = 132)	4.5 (*N* = 22)
Similar to previous ERS in-person congresses (Congress included: Live plenaries of experts’ presentations e.g., latest scientific advancements, clinical debates and case discussions)	15.7 (*N* = 79)	
Opportunities for interaction between all members (e.g., attendees, speakers, patients)	13.5 (*N* = 68)	8.6 (*N* = 42)
Clinical debate sessions	11.7 (*N* = 59)	6.6 (*N* = 32)
Clinical knowledge update sessions	9.3 (*N* = 47)	10.1 (*N* = 49)
Opportunities to interact with experts	6.8 (*N* = 34)	
Clinical case discussions	4.8 (*N* = 23)	19.8** (*N* = 96)
Poster and oral presentation sessions	4.8 (*N* = 24)	9.5** (*N* = 46)
Workshops facilitating interaction between attendees and speakers	3.8 (*N* = 19)	1.2 (*N* = 6)
Better access to simultaneously virtually stream the in-person conference at	2.5 (*N* = 13)	
Consideration of different time zones/languages	0.9 (*N* = 5)	
Similar to ERS virtual congress 2021 (Congress included: Live streaming online of experts’ presentations e.g., latest scientific advancements, clinical debates and case discussions)		24.3 (*N* = 118)
Recordings available for longer		6.8 (*N* = 33)
Increased variation of topics		3.3 (*N* = 16)
Expert speaker presentations		3.2 (*N* = 16)
Technical Improvements		2.1 (*N* = 10)

* Frequencies less than five were omitted.

** *p* < 0.05 according to chi-squared analysis.

### Means of improving a large-scale virtual conference

In addition to asking attendees for their preferences for the virtual and in-person component of hybrid conferences in general terms, we also asked attendees “What improvements would you suggest for this year’s virtual congress?” as a means of understanding how to improve virtual components of conferences. 58.6% (*N* = 482) of respondents were highly satisfied (assigned 6 or 7 on a 7-point scale) with the 2021 ERS virtual congress. The factors that drove that success were dominantly “quality of speakers and presenters” (as indicated by 67.8% (*N* = 558) of respondents), the “relevance of topics/content of sessions” (65.1%; *N* = 536), and “interactivity within sessions and audience participation” (38.5%; *N* = 317).

361 attendees commented on improvements they would prioritise. They primarily focussed on greater interaction between members in the virtual platform (23.3%, *N* = 84), technical improvements (22.4%, *N* = 81) and increased variation of topics (14.4%, *N* = 52). Table 2 demonstrates direct content analysis codes and their frequencies outlining ways of improving a large-scale virtual conference.

**Table 2:** Improvements attendees suggested for a large-scale virtual conference

	Large-scale virtual conference improvements (Total *N* = 361)
Code from directed content analysis	% (N)*
Opportunities for interaction between all members (e.g., attendees, speakers, patients)	23.3 (*N* = 84)
Technical Improvements	22.4 (*N* = 81)
Increased variation of topics	14.4 (*N* = 52)
Less simultaneous virtual sessions	11.4 (*N* = 41)
Recordings available for longer	9.7(*N* = 35)
Consideration of different time zones/languages	5.8 (*N* = 21)
Move to Hybrid form with in-person component	5.5 (*N* = 20)
Clinical skills sessions	4.2 (*N* = 15)
Lower costs	1.9 (*N* = 7)
Opportunities to interact with experts	1.4 (*N* = 5)

* Frequencies less than five were omitted.

## Discussion

Our respondents indicated that the majority of them would prefer future meetings to take place in a hybrid format, with virtual sessions spread throughout the year in addition to during the congress itself. In doing so, they identified aspects of conferences they would prioritise for both in-person and virtual components. For in-person components of hybrid conferences, respondents recommended prioritising increasing the number of clinical skills sessions and live plenaries of experts’ presentations (e.g., latest scientific advancements, clinical debates and case discussions and, opportunities for interaction between delegates). For virtual components of hybrid conferences, respondents similarly recommended prioritising live streaming of experts’ presentations, but they also suggested increasing the overall number of clinical case discussions and facilitating opportunities for virtual discussions with experts. Suggested improvements for a large-scale virtual conference include prioritising both interaction between participants (attendees, speakers, patients) and technical improvements. Cost remains a major barrier for in-person conference attendance in addition to the challenges associated with travel.

As organisers strive to offer conferences that enable learning, global accessibility, and flexibility, the preference of candidates to have virtual components take place during the meeting and throughout the year takes on great importance. The literature, however, suggests that segregating the community of people with interest in a subject area into those who attend traditional in-person conferences and those who attend virtual meetings should be avoided, for fear of creating subgroups rather than taking proper advantage of the full community’s inherent ability to broaden the conference’s diversity and strengthen social networks [23]. This highlights a need to focus on continuing with hybrid formats with the now improved clarity of what aspects should be included in the respective in-person and virtual components.

While cost will inevitably prevent some people from attending in-person conferences [24], delegates’ desires for hybrid meetings reinforces the inequity of holding meetings that are purely in-person; incorporation of virtual components during hybrid meetings might help to enable greater interaction between those with more and those with fewer resources.

Focussing more granularly, respondents suggested that practicing live clinical skills should be prioritised for the in-person component of hybrid conferences (26.2% compared to 4.5% for the virtual component of hybrid conferences), in addition to networking. The former could include use of bronchoscopes and practice of novel surgical incisions in a simulated setting to broaden skill development. Conferences that can provide such in-person live clinical skills sessions, with experts facilitating, appear likely to attract attendees by offering direct learning they can translate back to their local setting. Recent literature suggests that more virtual reality–based technology may be used to improve the use of hands-on workshops after virtual sessions to reinforce the concepts learned in lectures and during live operative demonstrations [15]. Whether or not that can be made as effective as learning during in-person meetings remains to be seen given that face-to-face meetings allow participants additional benefits of listening to information while observing the speaker’s body language, facial expressions, and gestures (i.e., cues that are often difficult to detect virtually, but improve the ability of people to communicate effectively [25]).

That said, our findings suggest that clinical case discussions are more valued as part of the virtual component of the hybrid conferences compared to the in-person component (19.8% compared to 4.8%), thus supporting the idea that knowledge (as opposed to skill development) should be the focus when conducting virtual sessions. Consistent with that observation is that poster and oral presentation sessions were more frequently mentioned for inclusion in the virtual component of hybrid conferences. Such may be preferred by attendees in a virtual setting because they create the opportunity to present one’s findings to support continuous professional development through improving presentation skills and acquiring mandatory CPD points. They also grant the opportunity for learning from other presentations within the designated session, perhaps from the convenience of home. It is important to keep in mind, however, that previous research has reported that it should not be assumed that conference goers are a homogenous group; rather, subgroups of attendees and their different motivations for attendance likely need taken into account [19].

With respect to the large-scale nature of the conference focused upon in this study, it is noteworthy that the results show that participants envisioned the *quality of speakers/presenters* and *relevance of topics/content of sessions* to be fundamental determinants of their satisfaction with virtual conferences. Those findings are similar to those shared by Rubinger et al. [6] in their discussion of how to maximise virtual meetings and conferences following a review of conference best practices (i.e., they drew particular attention to speaker quality). Attracting high quality speakers may be more feasible in a large-scale virtual conference because conference organisers usually require a budget to cover travel costs for those who are invited to in-person conferences; the finances freed up might be used to source the best experts within a field. To ensure they meet the needs of attendees, Rubinger et al. stress the importance of ensuring that speakers have appropriate support documents and template presentations that take into account what participants should take away from the presentations [6]. Our own prior research comparing virtual conferences with past in-person conferences [10] suggested that participants would like the opportunity for knowledge gain from conferences to extend beyond that of the conference days, effectively lengthening the meeting by providing preparatory and follow-up resources. For conference organisers, pre-reading material, take-away messages in a summary document or virtual multiple-choice questions to test knowledge before and after the conference may be beneficial for attendees by lengthening the timespan in which they engage in learning.

In any case, a dominant issue for respondents in this study was the importance of greater interaction between all members in the virtual platform and the need for technical improvements. This highlights that networking is a main priority for attendees even in virtual conferences although they would prioritise in-person networking opportunities when hybrid formats are used. When conferences must be run entirely online, virtual networking opportunities may be particularly important for younger members of the community (e.g., students who may not have access to the financial means to travel to large-scale international conferences but are able to join online).

### Strengths, limitations and future research

Strengths of this study include its large-scale survey design and utilising an international and multidisciplinary population that was forced to grapple with questions of conference priorities (the focus of the research) as a result of the constantly changing circumstances of the COVID-19 pandemic. Conducting this study after the second ERS virtual conference, that is, created considerable opportunity to gather experience-informed guidance for conference organisers who now need to determine how to proceed with meetings in the future. Through investigation of delegate preferences and barriers faced, we were able to identify inequities inherent in offering in-person formats alone. This information will help conference organisers increase the utility of their meetings for all attendees.

The limitations associated with our study include a low response rate (4.9%) despite the use of multiple follow-up reminders and a lottery incentive, as suggested by [26]. Concern deriving from that fact is lessened to a degree by the sample size being large and the demographics being similar to what is expected from the conference delegate population. Selection bias may still exist, however, given that, for example, respondents with greater technical prowess may have been more readily able to fill out the survey. More generally, the decision to recruit from the delegate list of a virtual meeting means that we are missing the perspectives of those who did not attend the conference because they do not value the learning/interaction that is on offer through virtual meetings. It is noteworthy, however, that only 11% of respondents expressed a preference for virtual meetings alone, suggesting that participants were not simply those who were particularly supportive of the format in which the ERS took place. Unfortunately, the conference is unable to provide demographics for the full set of delegates, making it impossible to judge the representativeness of our sample but we would note that the gender and geographic distribution are similar to that of previous years [19].

Future research should include investigation into what specific sessions attendees would like to see within in-person and virtual components of hybrid conferences (e.g., online flipped-classrooms, live simulation multi-disciplinary team sessions to tackle respiratory emergencies) as well as how structured virtual socialising is perceived by attendees and/or supervisors, experts and mentors.

## Conclusion

Our study has given light to conference organisers regarding how future hybrid conferences might best meet the preferences and priorities of attendees. Such conferences would ideally include (a) an in-person component focussed on live clinical skills sessions and networking and (b) a virtual component with sessions, throughout the year, focussing on speakers who are experts in their field and able to deliver good online teaching and learning on a variety of topics. By targeting this balance in a hybrid conference, organisers can enable the vital interaction between individuals during the in-person component (e.g., networking, viewing and improving on clinical skills) while enabling them to access virtual content at their convenience.

### Electronic supplementary material

Below is the link to the electronic supplementary material.

Supplementary Material 1

## Data Availability

The datasets used and/or analysed during the current study are available from the corresponding author on request.
