# Supplementary material for: Voices of conference attendees: how should future hybrid conferences be designed?
Source: BMC Med Educ. 2024 Apr 9;24:393. doi: 10.1186/s12909-024-05351-z (PMC11005117; doi:10.1186/s12909-024-05351-z)
Supplement: Supplementary file 1 — Supplementary Material 1 [file 12909_2024_5351_MOESM1_ESM.docx]

**Appendix 1**

**Part 1: Virtual ERS International Congress 2021**

1. How satisfied were you by the virtual ERS International Congress 2021 overall?

*(Closed question: Not at all satisfied (1) to Completely satisfied (7).)*

2. How many times have you attended an ERS International Congress in person?

*(Closed question: select one option from list below)*

Never

1

2-4

5-9

10-19

20 or more

3. Did you attend the virtual ERS International Congress 2020?

*(Closed question: Yes/No)*

4. Were there any barriers to previously attending ERS in-person congresses? (e.g., registration cost, travel distance, accommodation, language)? If yes, what were they? *(Free Text)*

5. Which format would you prefer in the future?

*(Closed question: select one option from list below)*

- Online

- In Person

- A combination of both

- No preference

**Part 2: Reasons for attending virtual ERS International Congress 2021**

6. Why did you take part in the virtual ERS International Congress 2021?

*(Closed question: Rate the statements below from strongly disagree (1) to strongly agree (7) or Not Applicable (N/A))*

I attended **this year’s virtual** **conference 2021**:

- **to support my career development**
- **to support career developments of others**
- **to socially interact and spend time with peers**
- **to present my scientific/academic work**
- **to meet experts and leaders in the field**
- **to make/deepen professional connections**
- **to learn the latest scientific findings**
- **to learn the latest advancements in patient care**
- **to improve my practical clinical skills** **(e.g., online live bronchoscopy procedure)**
- **to improve my communication skills (e.g., patient communication, team communication)**
- **to improve my teaching skills**
- **to fulfil the requirements of professional certification bodies, such as attaining CPD/CME credits**
- **to foster personal change**
- **to foster change in my workplace**

7. How well were the following reasons fulfilled by this year’s virtual conference?

*(Closed question: Rate the statements below from strongly disagree (1) to strongly agree (7) or Not Applicable (N/A))*

- **to support my career development**
- **to support career developments of others**
- **to socially interact and spend time with peers**
- **to present my scientific/academic work**
- **to meet experts and leaders in the field**
- **to make/deepen professional connections**
- **to learn the latest scientific findings**
- **to learn the latest advancements in patient care**
- **to improve my practical clinical skills** **(e.g. online live bronchoscopy procedure)**
- **to improve my communication skills (e.g., patient communication, team communication)**
- **to improve my teaching skills**
- **to fulfil the requirements of professional certification bodies, such as attaining CPD/CME credits**
- **to foster personal change**
- **to foster change in my workplace**

8a) Which sessions supported the reasons for your virtual conference attendance?

*(Closed question: Please select all options that apply)*

- **Challenging clinical cases**
- **Clinical trials session (ALERT)**
- **E-posters**
- **Expert view**
- **Grand rounds (case-based interactive sessions)**
- **Guideline session**
- **Hot topics**
- **Joint society session**
- **Journal session**
- **Language session**
- **Live from the clinic**
- **Lungs on fire**
- **Oral presentation (abstracts)**
- **Primary care session**
- **PRO/CON debate**
- **Professional development**
- **Respiratory medicine meets other disciplines**
- **State of the art**
- **Symposium**
- **Year in review**
- **None of the above**
- **Other (please specify)**

8b) How did these sessions support your reasons for attendance?

*(Free Text)*

9. Which aspects within this year’s **virtual** conference overall made it a success for you?

*(Closed question: Rate the statements below from strongly disagree (1) to strongly agree (7) or Not Applicable (N/A))*

- - **Interactivity during sessions/Audience participation**
  - **Relevance of topic/contents within sessions**
  - **Quality of Speakers/Presenters**
  - **Other (please specify- free text)**

**Part 3: Improvements and Future Conferences**

10. What improvements would you suggest for this year’s virtual congress?

(*Free Text*)

11. Would you come again to a similar virtual ERS Annual Congress (or recommend this one to colleagues)?

*(Closed question: select one option from list below)*

-No

-Possibly

-Yes

***We are thinking of moving to Hybrid conferences (combination of virtual and in-person components) for the future.***

12a) What would you like to see in the in-person component?

(*Free Text*)

b) What would you like to see in the virtual component?

(*Free Text*)

c) When should virtual sessions take place?

*(Closed question: select one option from list below)*

- **Several virtual sessions throughout the year**
- **Virtual sessions only during the in-person congress (in parallel)**
- **Combination of Both: virtual sessions during in-person congress and throughout the year**

**Part 4: Basic Demographic Data**

*For the final section, we would like to learn more about you and your professional role. Please kindly answer the few questions below.*

**Age:**

*(Closed question: select one option from list below)*

<20 21-25

26-30 31-35

36-40 41-45

46-50 51-55

56-60 61-65

66-70 >70

**Gender:**

*(Closed question: select one option from list below)*

Male

Female

Prefer not to say

**Country of Practice**:

*Drop-down menu*

**ERS Member:**

*(Closed question: select one option from list below)*

Yes

No

**Professional Category:**

*(Please select all that apply and give further details in “Other” section)*

Adult Pulmonologist/Clinician

Clinical Researcher

General Practitioner

Student (please specify area of study in “Other” section)

PhD Student (please specify area of study in “Other” section)

Nurses

Paediatrician

Physician in Pulmonary Training

Physiologist

Radiologist

Respiratory Critical Care Physician

Respiratory Physiotherapists

Respiratory Therapists

Thoracic Oncologist

Thoracic Surgeon

Educator e.g., lecturer

Journalist

Pathologist

Patient

Sales, Marketing, Industry

Scientist

Thoracic Oncologist

Thoracic Surgeon

Other (Free text)

**Please select at which career stage best suits your current role?**

*(Please select one option and give further details in “Other” section)*

- **Student** (e.g., medical student)
- **Early Career:** Junior Role (e.g., junior doctor, junior nurse)
- **Mid Career:**  Mid Level Role (e.g., respiratory registrar, senior physician in training)
- **Late Career:** Senior Clinical or Academic Role (e.g., consultant in respiratory medicine, professor)
- **Other role** (please specify) (*Free Text*)

**Place of Practice:**

*(Please select one option or give further details in “Other” section)*

Academic institution

University hospital

Non-university hospital

Private/Independent

Industry

Governmental organisation

Non-governmental organisation

Other……. *(Free Text)*

Thank you very much for your time and responses to help us shape future conferences!

**Appendix 2** Demographic Data for Survey Participants

| Respondents’ Age Ranges. (Years) | % | N |
| --- | --- | --- |
| 21-25 | 1.4 | 9 |
| 26-30 | 7.1 | 47 |
| 31-35 | 12.2 | 81 |
| 36-40 | 14.1 | 94 |
| 41-45 | 15.8 | 105 |
| 46-50 | 10.8 | 72 |
| 51-55 | 12.5 | 83 |
| 56-60 | 11.4 | 76 |
| 61-65 | 8.4 | 56 |
| 66-70 | 4.5 | 30 |
| >70 | 1.8 | 12 |
| Total | **100** | **665** |

**Appendix 3** Demographic Data for Survey Participants

| Respondents’ Place of Work | % | N |
| --- | --- | --- |
| Non-government Organisation | 1.4 | 9 |
| Other | 2.4 | 16 |
| Industry | 4.2 | 28 |
| Government Organisation | 6.5 | 43 |
| Private/Independent | 10.8 | 72 |
| Academic Institution | 17.1 | 114 |
| Non-university Hospital | 18.3 | 122 |
| University Hospital | 39.3 | 261 |
| Total | **100** | **665** |
|  |  |  |
